# Influence of Chinese university track-and-field coaches’ communication ability on student-athletes’ sport passion and ego-resilience

**DOI:** 10.3389/fpsyg.2026.1788617

**Published:** 2026-04-08

**Authors:** Xi Chen, Luping Cao, Ning Cai, Yi Zhang, Yunhang Lu

**Affiliations:** 1 School of Physical Education and Health Engineering, Taiyuan University of Technology, Taiyuan, China; 2 Department of Physical Education, Kyungpook National University, Daegu, Republic of Korea; 3 School of Physical Education and Sports Science, Soochow University, Suzhou, China

**Keywords:** coach communication competence, coach–athlete interaction, dualistic model of passion, ego-resilience, self-determination theory

## Abstract

**Background:**

This study examined the relationships between Chinese university track-and-field coaches’ communication ability, student-athletes’ sport passion, and ego-resilience. It further tested whether sport passion mediates the association between coaches’ communication ability and athletes’ ego-resilience.

**Methods:**

A questionnaire survey was applied to Chinese university track-and-field student-athletes, yielding 412 valid responses. Descriptive statistics were computed, and the psychometric properties of the measures were evaluated using exploratory factor analysis, confirmatory factor analysis, and reliability analysis. Pearson correlation analysis was conducted to examine bivariate associations. Subsequently, structural equation modeling was performed to test the hypothesized direct and indirect (mediated) pathways with model fit evaluated using commonly accepted fit indices.

**Results:**

The structural model demonstrated acceptable fit (χ^2^/df = 2.261, RMR = 0.020, RMSEA = 0.055, CFI = 0.978, TLI = 0.967). Coaches’ communication ability had significant positive effects on sport passion (*B* = 0.668, *p* < 0.001) and ego-resilience (*B* = 0.294, *p* < 0.001), and sport passion positively predicted ego-resilience (*B* = 0.552, *p* < 0.001). Sport passion significantly mediated the association between coaches’ communication ability and ego-resilience.

**Conclusion:**

Findings highlight the importance of coaches’ communication ability in fostering student-athletes’ adaptive psychological resources. Interventions aimed at improving coaches’ communication may help strengthen athletes’ sport passion and in turn support the development of ego-resilience.

## 1 Introduction

In recent years, collegiate track-and-field student-athletes have increasingly appeared on the international stage and delivered strong competitive performances. These achievements are typically pursued under a high-load dual-career routine in which intensive training and competition benchmarks must be sustained alongside substantial academic requirements (Jiang and Wang, 2025; Sun et al., 2023). For many student-athletes, these dual-career demands are primarily manifested as time conflicts between training and study, cumulative role strain, and structural constraints that can undermine sustained academic engagement and training continuity (Cosh and Tully, 2015; Stambulova and Wylleman, 2015). Because such pressures are rarely fully alleviated in the short term, athletes’ ability to maintain stable involvement and achieve adaptive psychological functioning may depend strongly on proximal, modifiable sources of support and regulation embedded in day-to-day training contexts (Knight et al., 2018; Petiot et al., 2024). This issue is particularly salient in track and field, where training and performance evaluation emphasize individualized outcomes; consequently, athletes’ access to training information, comprehension of technical feedback, and quality of self-regulation and persistence under stress tend to be shaped through routine, on-site coach–athlete interaction and communication (Pacewicz and Smith, 2023; Praça et al., 2022; Wilson et al., 2021). Accordingly, identifying key contextual elements in the training environment that are amenable to intervention and clarifying how these elements relate to student-athletes’ adaptation and mental and physical health have become central priorities in contemporary dual-career research and practice initiatives (Saarinen et al., 2025; Stambulova et al., 2024).

Within the training context, coaches are typically among the most influential social agents for student-athletes. Their impact extends beyond planning training content and managing training load; through the ways information is framed, feedback is delivered, and relationships are cultivated in everyday interactions, coaches can continuously shape athletes’ motivational experiences and emotional responses (Davis et al., 2019; Lu et al., 2024; Mageau and Vallerand, 2003; Petiot et al., 2025). Communication scholarship conceptualizes communicative competence as the capacity to engage in interaction that is effective and appropriate within a given relational context, and it is commonly articulated in terms of three core components: communication motivation, knowledge, and skills (Carmack and Harville, 2020; Lee et al., 2022). In sport settings, coaches’ interpersonal and interactional styles are often expressed through athletes’ perceived communication experiences and are linked to the quality of the coach–athlete relationship and to psychological adaptation outcomes. For instance, supportive interpersonal coaching styles have been associated with high levels of individual resilience among athletes, while high-quality coach–athlete relationships have been linked to low risk of athlete burnout (Fan et al., 2023; Isoard-Gautheur et al., 2016; Llanos-Muñoz et al., 2023). Hence, conceptualizing “coach communication competence” as a proximal factor embedded in daily training interactions—one that is trainable and amenable to intervention—not only enhances the practical utility of the theoretical account but also provides a clear leverage point for coach education and training-context interventions.

Self-determination theory posits that when individuals’ basic psychological needs for autonomy, competence, and relatedness are supported in a given activity context, they likely develop high-quality autonomous motivation and experience improved psychological functioning and well-being (Deci and Ryan, 2000; Klein, 2019). From this perspective, coach communication competence can be conceptualized as a salient contextual cue in training interactions: When coaches convey training goals and feedback in ways that are clear, respectful, and autonomy-supportive, athletes may experience strong competence and relatedness, which in turn facilitates the internalization of training values and promotes self-determined engagement (Deci and Ryan, 2000). Against this backdrop, sport passion has been defined as a strong inclination toward an important activity that is internalized into one’s identity and, as such, energizes sustained and stable involvement over time (Vallerand and Verner-Filion, 2020). The dualistic model of passion further distinguishes two internalization pathways: Harmonious passion, which reflects autonomous internalization, is typically associated with flexible engagement and adaptive outcomes; by contrast, obsessive passion, which is strongly driven by internal or external pressures, likely involves conflict experiences and potential psychological costs (Vallerand et al., 2003). Concurrently, ego-resilience refers to the capacity to maintain flexible self-regulation and recover relatively quickly under changing situational demands and stress, representing an important psychological resource for student-athletes navigating fluctuations in training and academic requirements (Liu et al., 2016; Paquette et al., 2023). In the broader mental health literature, high resilience is generally associated with low levels of negative psychological symptoms, including depression and anxiety (Wen et al., 2023). Accordingly, in dual-career contexts in which student-athletes routinely face concurrent demands from sport and education, examining how modifiable factors in the training environment relate to resilience development through motivational experiences can provide targeted empirical evidence to inform dual-career support and mental health promotion efforts (Stambulova et al., 2024).

Recent work—often grounded in self-determination theory—has repeatedly shown that coaches’ autonomy support and the quality of the coach–athlete relationship are closely associated with athletes’ motivational experiences and adaptation outcomes (Dong et al., 2024; Kim and Choi, 2024). However, evidence at the microlevel of day-to-day training interactions remains comparatively underdeveloped. In particular, prior research has frequently summarized “coach influence” in broad terms such as supportive versus controlling orientations, leaving an explanatory question insufficiently addressed: Through which specific, trainable communication behaviors (e.g., explaining, listening, providing feedback, and negotiating) do coaches translate technical information, task demands, and psychosocial support into motivational cues and coping resources as perceived by athletes? Related evidence suggests that training communication and athletes’ subjective communication experiences may play a key role in linking coach behaviors to athletes’ psychological outcomes (Choi et al., 2020; Järdmo et al., 2023). The dualistic model of passion provides a fine-grained motivational framework for understanding qualitative differences in sustained engagement, and recent studies have used this model to explain how passion relates to adaptive resources such as resilience (Ancarani et al., 2024). In the context of growing attention on dual-career demands and the increasing emphasis on contextual support (Stambulova et al., 2024), the present study draws on self-determination theory and the dualistic model of passion to develop and test a structural equation model (SEM) among Chinese university track-and-field student-athletes. Specifically, we examine the direct associations of coaches’ communication competence with sport passion and ego-resilience, and we further test the indirect association of coaches’ communication competence with ego-resilience via sport passion. In doing so, this study aims to provide targeted empirical evidence to inform communication-focused coach development and psychological adaptation support in training settings. The following hypotheses are proposed:

*Hypothesis 1*: Coaches’ communication competence has a significant positive effect on student-athletes’ sport passion.

*Hypothesis 2*: Student-athletes’ sport passion exerts a significant positive effect on their ego-resilience.

*Hypothesis 3*: Coaches’ communication competence has a significant positive effect on student-athletes’ ego-resilience.

*Hypothesis 4*: Student-athletes’ sport passion mediates the relationship between coaches’ communication competence and ego-resilience.

## 2 Methods

### 2.1 Participants

This study recruited student-athletes from high-performing university track-and-field teams across mainland China. A nonprobability sampling approach was used, combining purposive recruitment of high-performing university teams with convenience-based participation subject to team access and scheduling. Data collection was launched in Jiangsu, Shanxi, and Liaoning provinces and then scaled up via administrator referrals to additional universities nationwide. Eligible institutions were those that offer track-and-field programs and operate varsity teams under the high-level athlete admission (sport specialty recruitment) system. In sum, participants were recruited from 28 universities across 6 provinces. A total of 426 questionnaires were returned; after data-quality screening, 412 were retained for analysis (valid response rate = 96.7%). The final sample comprised 259 men (62.9%) and 153 women (37.1%), with a mean age of 21.4 years (SD = 2.10). Most participants were undergraduates (92.2%), and the largest proportion reported over 4 years of training experience (40.0%). The most common training frequencies were three and five sessions per week (both 28.4%). Session duration was typically 1–2 h (52.9%) or 2–3 h (33.7%). Participants primarily competed in track events (44.4%) and field events (35.4%). Provincial-level awards were the most frequently reported (41.7%). Coaches in the participating teams had no formal education or certification in sport psychology or related psychological training, and the teams did not have a designated sport psychologist integrated into their regular training program. Table 1 presents the detailed demographic and training.

**Table 1:** Demographic characteristics of the research subjects.

Category		*N*	%
Sex	Male	259	62.9
	Female	153	37.1
Age (years)	<18	11	2.7
	19	49	11.9
	20	85	20.6
	21	80	19.4
	22	80	19.4
	23	43	10.4
	24	24	5.8
	≥25	40	9.7
Academic year	Undergraduate, Year 1	60	14.6
	Undergraduate, Year 2	115	27.9
	Undergraduate, Year 3	104	25.2
	Undergraduate, Year 4	90	21.8
	Undergraduate, Year 5	11	2.7
	Graduate, Year 1	8	1.9
	Graduate, Year 2	10	2.4
	Graduate, Year 3	14	3.4
Admission pathway	General admission (sport-related track)	232	56.3
	Independent sport admission	61	14.8
	High-level athlete/specialty admission	119	28.9
Years of training	≤1	39	9.5
	2	85	20.6
	3	78	18.9
	4	45	10.9
	>4	165	40
Training frequency (sessions/week)	≤2	24	5.8
	3	117	28.4
	4	50	12.1
	5	117	28.4
	6	66	16
	≥7	38	9.2
Event type	Field events	146	35.4
	Track events	183	44.4
	Both track and field	83	20.1
Training duration (hours/session)	<1	21	5.1
	1–2	218	52.9
	2–3	139	33.7
	3–4	27	6.6
	>4	7	1.7
Academic major	Sport training	189	45.9
	Physical education	134	32.5
	Other majors	89	21.6
Awards	None	59	14.3
	University-level	70	17
	City/municipal-level	56	13.6
	Provincial-level	172	41.7
	National-level	47	11.4
	International-level	8	1.9
University type	Comprehensive university	395	95.9
	Sport university	17	4.1
Total		412	100

### 2.2 Measures

This study used self-report questionnaires to assess coaches’ communication ability, sport passion, and ego-resilience. All items were rated on a 5-point Likert scale (1 = strongly disagree, 5 = strongly agree), with higher scores indicating higher levels of the corresponding psychological constructs.

#### 2.2.1 Coach–athlete communication

Coach–athlete communication was assessed using the coach communication competence Scale (Fritz et al., 1999). On the basis of the original instrument, we consulted the Korean translation by Choi (2010) and incorporated revised items reported by Choi (2012) and Lee (2011), with minor adaptations to reflect the context of university track-and-field training in China. The scale captures student-athletes’ perceptions of their coaches’ communication competence. Following exploratory factor analysis and confirmatory factor analysis (CFA), the final version retained 2 dimensions with 24 items: Response and Analysis and Understanding and Memory. Internal consistency indices for the total scale and subscales are reported in Table 2.

**Table 2:** Reliability of coach communication competence, sport passion, and ego-resilience.

Construct	Subscale	Cronbach’s α (subscale)	Cronbach’s α (overall)
Coach communication competence (CCA)	Response and analysis (Items 1, 3–17)	0.974	0.969
	Understanding and memory (Items 18–25)	0.907	
Sport passion (SP)	Harmonious passion (Items 1–6)	0.895	0.944
	Obsessive passion (Items 7–14)	0.950	
Ego-resilience (ER)	Emotional regulation (Items 11–20, 25)	0.937	0.942
	Positive thinking (Items 1–5, 10)	0.931	
	Goal orientation (Items 21–24, 30, 31)	0.869	
	Negative emotion (Items 6–8)	0.890	
	Communication style (Items 28, 29, 32)	0.663	

Cronbach’s α indicates internal consistency reliability. Item numbers refer to the final retained items.

#### 2.2.2 Sport passion

Student-athletes’ sport passion was assessed using the Passion Scale (Vallerand et al., 2003). We also consulted prior reconstructed/revised versions (Koo, 2012; Lim, 2008) to guide item selection and wording refinement for the present context. The scale comprises 14 items and 2 subscales: Harmonious Passion (6 items) and Obsessive Passion (8 items). In the current sample, Cronbach’s α was 0.895 for Harmonious Passion and 0.950 for Obsessive Passion, with an overall reliability of 0.944 (Table 2).

#### 2.2.3 Ego-resilience

Ego-resilience was measured using the ego-resilience Scale for University Students (Lee and Kim, 2012). The scale was used to assess student-athletes’ ego-resilience. The final version retained 29 items across 5 dimensions: Emotional Regulation, Positive Thinking, Goal Orientation, Negative Emotion, and Communication Style. In the present sample, Cronbach’s α coefficients for the five dimensions were 0.937, 0.931, 0.869, 0.890, and 0.663, respectively; the overall reliability was 0.942 (Table 2).

### 2.3 Procedure

Data were collected using a combination of online and in-person approaches. Specifically, data were acquired from June 2024 to May 2025 via site-by-site survey administration across participating universities, using QR code on-site and online remote completion as needed. The research team first held remote meetings with the administrators of university track-and-field teams to introduce the study and coordinate the survey schedule. After obtaining permission from the team administrators, the research team conducted on-site visits and distributed the questionnaires. The questionnaire package was constructed by integrating the finalized versions of the study measures and a brief demographic/training form, and it was delivered in a standardized order to ensure consistency across sites. Before participation, investigators provided standardized instructions regarding the study purpose, response procedures, and participants’ rights, emphasizing that participation was entirely voluntary and that participants could refuse to answer any question or withdraw at any time without penalty. The study protocol was reviewed and approved by the Ethics Committee of Soochow University (Approval No.: SUDA20250609H19). All participants received full study information and provided written informed consent (signed in person or confirmed electronically) prior to completing the questionnaire, and only those who consented were enrolled and allowed to proceed with the questionnaire.

The survey was applied as a self-applied questionnaire. Participants completed the questionnaire independently by scanning a QR code, which typically required approximately 10 min. Investigators responded only to participants’ questions on item comprehension without providing guidance that could influence responses. After data collection, responses were screened using prespecified data-quality criteria (e.g., excluding invalid submissions and cases with abnormal completion times) to generate the final dataset for statistical analyses.

No directly identifiable personal information was collected. All data were recorded and stored anonymously and were used solely for statistical analysis and academic dissemination. Results were reported in aggregate to protect participant privacy and ensure data security. After completing the questionnaire, participants received a small gift as a token of appreciation.

### 2.4 Data analysis

All statistical analyses were conducted using SPSS 25.0 and AMOS 23.0. First, descriptive statistics were computed to summarize participants’ demographic and training characteristics (frequencies, percentages, means, and standard deviations). Second, Pearson product–moment correlations were used to examine zero-order associations among the study variables. Moreover, the range of correlation coefficients was inspected to preliminarily assess the risk of multicollinearity.

For measurement model evaluation, exploratory factor analysis (EFA) was first performed to examine the latent structure of the scales in the present sample; items were screened on the basis of factor loadings and cross-loadings. Subsequently, confirmatory factor analysis (CFA) was conducted in AMOS using the maximum likelihood estimator. Model fit was evaluated using χ^2^/df, RMR, RMSEA, CFI, TLI, and parsimonious fit indices (PNFI and PGFI). Reliability was assessed using Cronbach’s α and composite reliability (CR), and convergent validity was evaluated using the average variance extracted (AVE).

After acceptable measurement model fit was established, a SEM was specified to test the hypothesized pathways. The hypothesized structural model was tested in AMOS 23.0 using the maximum likelihood estimator. A conventional two-tailed significance level of *p* < 0.05 was adopted. Model error and overall fit were evaluated using χ^2^/df, RMR, RMSEA, CFI, TLI, and parsimonious fit indices (PNFI and PGFI). Unstandardized path coefficients (B) and their statistical significance were reported. Indirect (mediation) effects were examined using bootstrapping to estimate confidence intervals, with mediation considered significant when the 95% confidence interval did not include zero.

## 3 Results

### 3.1 Correlation analysis

Pearson correlations among coach communication competence, sport passion, and ego-resilience are presented in Table 3. All correlations were below the commonly used threshold for potential multicollinearity (*r* > 0.85), indicating no evidence of severe multicollinearity in the present data (Kline, 2023). Accordingly, no severe multicollinearity existed in this study. Table 3 reports the significance levels for all correlations.

**Table 3:** Pearson correlations among study variables.

Variable	1	2	3	4	5	6	7	8	9
Response and analysis	1								
Understanding and memory	0.705**	1							
Harmonious passion	0.458**	0.401**	1						
Obsessive passion	0.513**	0.415**	0.632**	1					
Emotional regulation	0.459**	0.366**	0.447**	0.437**	1				
Positive thinking	0.402**	0.398**	0.425**	0.435**	0.384**	1			
Goal orientation	0.404**	0.360**	0.357**	0.430**	0.439**	0.568**	1		
Negative emotion	0.400**	0.313**	0.371**	0.450**	0.491**	0.429**	0.515**	1	
Communication style	0.266**	0.266**	0.187**	0.252**	0.322**	0.262**	0.351**	0.298**	1

**p < 0.01, *p < 0.05.

### 3.2 Measurement model evaluation

CFA was conducted to evaluate the measurement validity of the study variables. The two-factor model of coach communication competence showed adequate fit (χ^2^/df = 2.850, RMSEA = 0.067, CFI = 0.954, TLI = 0.950). The five-factor model of ego-resilience also demonstrated good fit (χ^2^/df = 2.487, RMSEA = 0.060, CFI = 0.935, TLI = 0.928). The two-factor model of sport passion likewise showed acceptable fit (χ^2^/df = 2.850, RMSEA = 0.067, CFI = 0.954, TLI = 0.950). Internal consistency reliability (Cronbach’s α) for each scale and subscale is reported in Table 2. In addition, CR and AVE supported adequate convergent validity (AVE ≥ 0.50). Overall, these results indicate acceptable measurement properties and provide a basis for subsequent structural model testing.

### 3.3 Structural model testing

**Figure 1:**
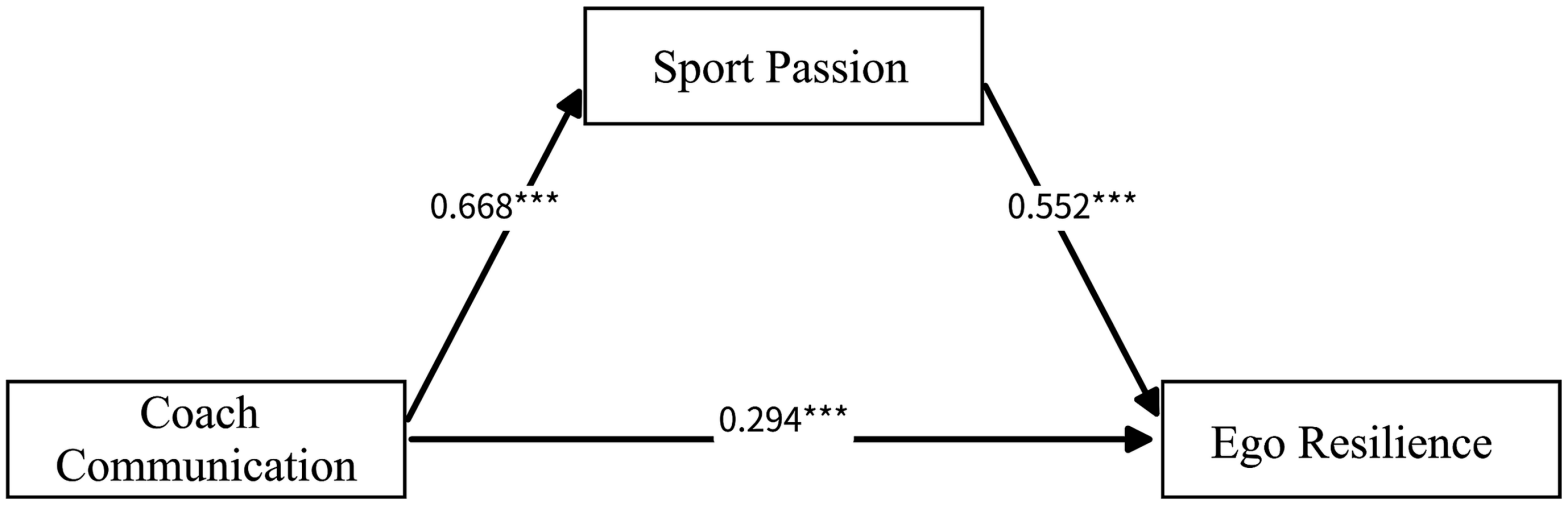
Relationships among variables in the proposed model. Values represent unstandardized path coefficients (B); ***p < 0.001.

After acceptable measurement model fit was established, a SEM was specified to test the hypothesized pathways. The structural model demonstrated acceptable overall fit to the data (Table 4). As hypothesized, coach communication competence positively predicted sport passion, sport passion positively predicted ego-resilience, and coach communication competence showed a significant direct association with ego-resilience. Collectively, these findings supported Hypotheses 1–3. Detailed parameter estimates are reported in Table 5, and the model is presented in Figure 1.

**Table 4:** Model fit indices for the structural model.

Fit index	χ^2^/df	RMR	RMSEA	GFI	NFI	TLI	CFI	PGFI	PNFI
Observed value	2.261	0.020	0.055	0.971	0.962	0.967	0.978	0.518	0.641
Recommended criterion	≤3	≤0.08		≥0.90				≥0.50	

χ^2^/df, chi-square/degrees of freedom; RMR, root mean square residual; RMSEA, root mean square error of approximation; GFI, goodness-of-fit index; NFI, normed fit index; TLI, Tucker–Lewis index; CFI, comparative fit index; PGFI, parsimony goodness-of-fit index; PNFI, parsimony normed fit index.

**Table 5:** Structural model results (unstandardized path coefficients).

Path	*B*	S. E.	C. R.	Hypothesis
Coach communication competence → sport passion	0.668	0.077	11.158***	Supported
Sport passion → ego-resilience	0.552	0.063	6.498***	Supported
Coach communication competence → ego-resilience	0.294	0.071	3.936***	Supported

*B* indicates the unstandardized path coefficient. S. E., standard error; C. R., critical ratio. ***p < 0.001.

### 3.4 Mediation analysis

To examine whether sport passion mediates the association between coach communication competence and student-athletes’ ego-resilience, we used a bootstrap procedure to test the mediation model. As shown in Table 6, bootstrapping indicated a significant indirect effect of coach communication competence on ego-resilience through sport passion, with the confidence interval not including zero. The direct effect remained significant after accounting for sport passion, suggesting a partial mediation pattern. Overall, the indirect pathway explained more than half of the total effect. Therefore, Hypothesis 4 was supported.

**Table 6:** Testing the mediating effect of sport passion on the relationship between coach communication competence and student-athletes’ ego-resilience.

Model effect	Effect size	Boot SE	95% CI		*P*
			LLCI	ULCI	
Total effect	0.663	0.048	0.523	0.702	<0.001
Direct effect	0.294	0.076	0.141	0.437	0.004
Indirect effect	0.369	0.064	0.263	0.527	0.001

Boot SE, bootstrap standard error; 95% CI, bootstrap confidence interval; LLCI and ULCI indicate the lower and upper limits of the confidence interval, respectively.

## 4 Discussion

This study used data from 412 Chinese university track-and-field student-athletes to systematically examine the relationships among coaches’ communication competence, sport passion, and ego-resilience. The structural model indicated that coaches’ communication competence had significant positive effects on sport passion (*B* = 0.668, *p* < 0.001) and ego-resilience (*B* = 0.294, *p* < 0.001) and that sport passion, in turn, significantly and positively predicted ego-resilience (*B* = 0.552, *p* < 0.001). Bootstrapped mediation analyses further showed that sport passion partially mediated the association between coaches’ communication competence and ego-resilience, with a significant indirect effect of 0.369 (95% CI [0.263, 0.527]), while the direct effect remained significant. Taken together, these findings support the proposed hypotheses and suggest that high-quality coach communication may be directly linked to student-athletes’ adaptive psychological resources and promote the accumulation and development of ego-resilience indirectly by activating and sustaining sport passion.

### 4.1 Coach communication competence and sport passion

Coaches’ communication competence significantly and positively predicted athletes’ sport passion (*B* = 0.668, *p* < 0.001), thereby supporting Hypothesis 1. This finding suggests that the function of communication extends beyond the transmission of training information; rather, it may shape athletes’ internal engagement by providing salient motivational cues. Specifically, when coaches clearly articulate training goals and their underlying meaning and respond to athletes’ feedback through attentive listening and constructive dialog, athletes likely internalize the value of training, experience great satisfaction of autonomy and competence needs, and consequently report high levels of sport passion (Deci and Ryan, 2000). This result is consistent with that from prior work showing that high-quality coach–athlete relationships are associated with great enthusiasm and motivation for sport participation (Lafrenière et al., 2008). Importantly, the effects of communication may depend on its interactional orientation: When communication is directive, controlling, or coercive, athletes’ autonomy need satisfaction may be undermined, attenuating the positive influence of communication on passion (Morbée et al., 2024).

### 4.2 Sport passion and ego-resilience

The results indicated that sport passion significantly and positively predicted ego-resilience (*B* = 0.552, *p* < 0.001), thereby supporting Hypothesis 2. This finding suggests that passion may function as a relatively stable motivational and affective investment resource that facilitates athletes’ persistence and recovery under stressful conditions. Prior research shows that high levels of passion are typically associated with great experiences of positive emotions and adaptive outcomes (Paquette et al., 2023). Positive emotions, in turn, may broaden individuals’ cognitive and behavioral repertoires, enabling fast recovery following setbacks and supporting flexible self-regulation (Fredrickson, 2004). In the present study, passion was modeled as a global construct within the structural model. Accordingly, the current findings robustly support a positive association between overall passion and resilience, but they do not allow for a clear differentiation of the effects of distinct passion types. Based on the dualistic model of passion, harmonious passion is consistently linked to positive adjustment, whereas obsessive passion may be associated with psychological costs such as anxiety and burnout (Curran et al., 2015; Vallerand, 2016). Therefore, the positive effect observed in this study may primarily reflect the role of harmonious passion. Inconsistencies in prior evidence regarding the passion–adjustment relationship may be attributable to differences in athletes’ stress levels, contextual characteristics, or measurement focus.

### 4.3 Coach communication competence and ego-resilience

Beyond the indirect pathway via passion, the influence of coach communication on athletes’ resilience does not appear to rely exclusively on this mechanism. The results showed a significant direct path from coach communication competence to ego-resilience (*B* = 0.294, *p* < 0.001), supporting Hypothesis 3 and suggesting that communication itself may operate as a relatively independent, contextual support resource. When coaches provide clear, actionable feedback and guidance and convey understanding and care in their interactions, athletes likely develop a strong sense of control over the training environment and great relational trust. These resources may help athletes maintain psychological stability and adjust quickly when facing stressors or setbacks. This interpretation is consistent with the sport resilience literature, which emphasizes that supportive relationships and social support can directly buffer the adverse effects of stress and facilitate recovery and adaptation (Sarkar and Fletcher, 2014). In the context of collegiate track-and-field training in China, coaches often constitute one of athletes’ most central sources of support; thus, effective communication may be directly associated with resilience by strengthening perceived relational security and supportive experiences. Nonetheless, because the present study relied on cross-sectional self-report data, the causal direction of the direct path should be interpreted with caution. Athletes with high resilience may be inclined to evaluate coach communication positively. Moreover, single-source measurement may introduce common method bias, potentially inflating the magnitude of the estimated paths to some extent (Podsakoff et al., 2003). Therefore, the direct effect is more appropriately interpreted as evidence of a structural association rather than as a definitive causal conclusion.

### 4.4 The mediating role of sport passion

To further clarify the mechanism through which coach communication competence relates to ego-resilience, we examined sport passion as a mediator and found that it carried a significant portion of this association (indirect effect = 0.369, 95% CI [0.263, 0.527]). Meanwhile, the direct link between communication competence and ego-resilience remained significant, indicating partial mediation. This pattern is theoretically meaningful because it suggests that communication competence is not merely an interpersonal “nice-to-have” but a functional contextual input that reorganizes athletes’ motivational experience in ways that are consequential for adaptive functioning (Petiot et al., 2024). From a self-determination theory perspective, communication that is clear, timely, and autonomy-supportive can reduce ambiguity about goals and standards, transform external demands into personally endorsed commitments, and reinforce athletes’ perceived competence through informational feedback (Mageau and Vallerand, 2003; Ryan and Deci, 2020), In this sense, sport passion is not a redundant motivational label but an observable consolidation of internalized value and sustained energetic investment: When athletes experience their training as meaningful and self-endorsed, passion becomes stable, effort is minimally dependent on external pressure, and setbacks are likely processed as task-relevant signals rather than threats to self-worth. This motivational configuration plausibly supports ego-resilience by enabling flexible self-regulation under strain—maintaining persistence while adapting strategies, sustaining engagement while recalibrating expectations, and recovering efficiently from performance fluctuations. The mediating role of passion is also coherent with the broaden-and-build proposition that positive motivational and affective experiences broaden coping repertoires and facilitate the accumulation of enduring cognitive and emotional resources that strengthen adaptation capacity (Stanley and Schutte, 2023). Crucially, partial mediation indicates that coach communication competence contributes to ego-resilience through pathways beyond motivation. Even when sport passion is held constant, competent communication can strengthen resilience by shaping athletes’ day-to-day appraisal of stressors—interpreting training difficulties in performance-relevant terms, converting setbacks into actionable guidance that enhances perceived control, and maintaining predictable expectations through timely responsiveness—thereby reducing maladaptive uncertainty and supporting adaptive coping when competitive and academic pressures peak (Thatcher and Day, 2008). Taken together, sport passion functions as a key motivational conduit linking communication to ego-resilience, while communication competence operates as an independent, resilience-relevant contextual resource that supports adaptation under pressure (Sarkar and Fletcher, 2014).

### 4.5 Theoretical implications

The theoretical contribution of this study lies in the interpretation of coach communication competence within an integrative framework that bridges self-determination theory and sport resilience research. First, this study conceptualized communication as a form of contextual support input, and the findings indicate that it is associated with the internalization of motivation. This study provides a concrete, behaviorally grounded entry point for applying self-determination theory to coach–athlete interactions (Bengtsson et al., 2024; Ryan and Deci, 2020). Second, the mediating role of sport passion offers mechanism-level evidence for how social interaction may be translated into adaptive psychological resources, suggesting that communication may facilitate athletes’ recovery and adaptation under pressure by activating and sustaining stable internal investment (Stanley and Schutte, 2023). In addition, the findings underscore the theoretical significance of passion quality. Future research should differentiate harmonious and obsessive passion and examine whether different communication orientations yield differential adaptive outcomes through distinct passion types, thereby further specifying the motivation–resilience mechanism and its boundary conditions (Fasey et al., 2021). Finally, longitudinal or intervention designs with multisource assessments are recommended to test directional effects rigorously and reduce same-source bias.

Conceptually, this study incorporates coach communication and athletes’ psychological adaptation into a unified explanatory framework, emphasizing that communication is not merely a technical component of training management but may also foster the development of adaptive psychological resources through motivational and affective processes (Llanos-Muñoz et al., 2023). These findings point to an integrative explanatory pathway linking coach communication, sport passion, and ego-resilience, and further suggest that the quality of athletes’ passion may influence whether this motivational process translates into adaptive psychological outcomes. Moving forward, research should employ longitudinal or intervention designs and incorporate multisource assessments to strengthen causal inference and mitigate common method bias (Podsakoff et al., 2024).

## 5 Conclusion

Guided by the aim of clarifying how coaches’ communication competence relates to sport passion and ego-resilience among collegiate track-and-field student-athletes, this study developed and tested a coach communication competence–sport passion-ego-resilience model. The results indicated that coach communication competence was positively associated with sport passion and ego-resilience and that sport passion was positively associated with ego-resilience. Moreover, sport passion partially mediated the association between coach communication competence and ego-resilience, suggesting that coach communication may contribute to resilience directly and indirectly through athletes’ motivational processes. Theoretically, by integrating motivational and resilience perspectives, these findings offer a clear mechanistic account of how coach–athlete interactions may translate into athletes’ adaptive psychological resources via motivational functioning, and they provide empirical support for linking SDT-informed explanations of social-context effects on internalization with contemporary sport-resilience frameworks (Gupta and McCarthy, 2022; Mossman et al., 2024; Ryan and Deci, 2020). Practically, the results underscore communication competence as a core coaching competency in collegiate high-performance training: Rather than relying exclusively on training control and outcome pressure, clear goal explanations and actionable feedback, attentive listening and responsiveness, and a supportive interpersonal approach may foster sustained training engagement and positive motivational experiences, thereby facilitating the accumulation and development of ego-resilience.

### 5.1 Limitations and future research directions

This study has several limitations that should be addressed in future research. First, we used a cross-sectional survey design. Although this approach can test structural associations among variables, it cannot establish temporal ordering or clarify the direction of effects among coach communication, sport passion, and ego-resilience; therefore, interpretation of these relationships requires caution. Second, the data were primarily obtained from athletes’ self-reports, which may be influenced by common method bias and socially desirable responding, potentially inflating the observed associations. Third, the sample was drawn from the specific context of collegiate track-and-field training in China. Sport-specific characteristics and cultural factors may shape coaches’ communication practices and their psychological effects; thus, the generalizability of the findings should be further examined across other sports and cultural contexts. Fourth, sport passion was modeled as a global construct, which does not allow the differential effects of harmonious versus obsessive passion to be disentangled. Accordingly, the role of “passion quality” in resilience development warrants fine-grained investigation.

Building on these limitations, future studies could advance this line of research in several ways. First, longitudinal or experimental/intervention designs could be employed to test directionality and strengthen causal inference. Second, multisource and multimethod data could be incorporated to reduce systematic bias associated with single-source measurement. Third, theoretical models should further differentiate communication orientations and passion types to precisely explain how different communication approaches relate to distinct resilience outcomes through specific motivational processes. Fourth, the model should be replicated in a broader range of sports, populations, and cultural settings while identifying contextual factors that may moderate the observed relationships, thereby enhancing the explanatory scope and practical generalizability of the conclusions.

## Data Availability

The raw data supporting the conclusions of this article will be made available by the authors, without undue reservation.
